# A Case of Persistent Generalized Retrograde Autobiographical Amnesia Subsequent to the Great East Japan Earthquake in 2011

**DOI:** 10.1155/2017/5173605

**Published:** 2017-03-01

**Authors:** Yuji Odagaki

**Affiliations:** Department of Psychiatry, Faculty of Medicine, Saitama Medical University, 38 Morohongo, Moroyama-machi, Iruma-gun, Saitama 350-0495, Japan

## Abstract

Functional retrograde autobiographical amnesia is often associated with physical and/or psychological trauma. On 11 March 2011, the largest earthquake on record in Japan took place, and subsequent huge tsunami devastated the Pacific coast of northern Japan. This case report describes a patient suffering from retrograde episodic-autobiographical amnesia for whole life, persisting for even more than five years after the disaster. A Japanese man, presumably in his 40s, got police protection in April 2016 but was unable to respond to question about his own name. He lost all information about his personal identity, and his memory was wholly lost until the disaster on 11 March 2011. He was able to recall his life after the disaster, and semantic memories and social abilities were largely preserved. A medical examination performed on 1 November 2016 verified that he was awake, alert, and oriented to time, place, and person (except for himself). General physical and neurological examinations revealed no pathological findings. He also experienced some symptoms associated with posttraumatic stress disorder (PTSD), such as intrusive thoughts, flashbacks, and nightmares. No abnormalities were detected by biochemical test and brain magnetic resonance imaging (MRI). Physicians and other professionals who take care of victims of disaster should be aware of dissociative spectrum disorders, such as psychogenic amnesia.

## 1. Introduction

Functional amnesia involving autobiographical memory is a pathognomic sign in a major class of mental illnesses known as dissociative (conversion) disorders [1], with sudden onset subsequent to physical trauma and/or psychologically stressful events, such as natural disasters (e.g., earthquakes and floods), marital discord, physical assault, personal threats, and war or military-related activities [2]. On 11 March 2011, the largest earthquake on record in Japan with a magnitude of 9.0 on the Richter scale took place, and subsequent huge tsunami devastated the Pacific coast of northern Japan. Thousands of people experienced fear of death. Herein, a patient with retrograde episodic-autobiographical amnesia for whole life lasting for more than five years after the disaster is reported.

## 2. Case Report

A Japanese man, presumably in his 40s, visited our outpatient clinic of the Department of Psychiatry, Saitama Medical University Hospital, on 1 November 2016, accompanied by a caseworker of the regional welfare office.

The caseworker explained that the chief purpose of his visit was to be diagnosed officially by a psychiatrist in order to make a resident card issued to him. According to the caseworker, inhabitants called the police in April 2016, because they felt uncanny impression when they found a stranger wandering about aimlessly in haggard and exhausted appearance. He was questioned by a policeman, but he was unable to respond to question about his own name. He was protected by police and then under the guardianship of the regional welfare office as a person with global amnesia. He was given a fictitious name and birthday and had stayed at a public lodging house for half a year. The residential division of the government office needed a certificate that he lost his whole life memory in order to issue a new resident card to him.

Careful medical examination performed throughout an hour and a half resulted in the following findings. The patient was a scrawny man of medium height, with an impression of sincere and honest soul. His memory was wholly lost until 11 March 2011, when he found himself lying face down under a broken wall of a house. He was helped out of the heap of wreckage by neighbors, and he followed other sufferers from the disaster shouting “Run away! Tsunami coming!” for his life. He recognized that a great earthquake happened. It was uncertain whether he lost consciousness under the heap of wreckage, but the surrounding landscape was totally unfamiliar to him, and he realized that he could recall nothing about himself. He stayed in a shelter for several days, where he noticed that the place was named Rikuzentakata, Iwate prefecture, one of the most extensively damaged municipalities by the disaster. He was seen by a psychiatrist or psychologist of the rescue team who gave him a medical certificate indicating his global amnesia. Because he felt nothing around him was familiar to him and the shelter became too crowded with sufferers, he started to move to other shelters one by one. In all shelters in which he stayed, however, he found no acquaintance and nobody knew him. Finally, he went out of a shelter and started his long journey for five years to south on foot. He was hired as a day laborer engaged in restoration construction and lodged at all-hours Internet cafes. He saved some money and at last arrived at Kumagaya, a city located in northern part of Saitama prefecture, where he fell victim to a robbery. The robber extorted his wallet containing all money and the medical certificate from him and brought him by automobile to mountain solitudes, where he was released. He was wandering mountains for several days without food until the above-mentioned protection by police.

The police office made inquiries about him to the government offices in the area where he was attacked by the earthquake, resulting in no hit. The patient leads an austere and regular life on welfare. He is still unable to face up to TV program with a picture of earthquake or tsunami. When earthquakes, even if very slight, happen to him, he shudders with horror and suffers from a nightmare.

General physical and neurological examinations revealed no pathological findings. Although systematic and complex neuropsychological tests were not performed, his intelligence appeared to be within normal limits. He was awake, alert, and oriented to time, place, and person (except for himself), and he was able to respond correctly to all questions of the Abbreviated Mental Test (AMT) [3] except for the two items concerning his identity (i.e., “Age” and “Date of birth”). Short-term memory was intact, and he was easily able to recall his personal life episodes subsequent to the time when he became conscious on the chaotic ground on 11 March 2011. He has completely lost his autobiographical memory for his whole life until then. He had been anxious about his amnesia and desired to clarify his personal identity.

Laboratory biochemical data revealed no abnormalities. Brain magnetic resonance imaging (MRI) was also normal.

## 3. Discussion

This paper describes a patient who has been suffering from a generalized retrograde amnesia for whole life which was supposedly subsequent suddenly to the disaster caused by huge earthquake and has persisted more than five years. His memory impairment is limited to episodic-autobiographical memory system [4], and semantic memories and social abilities were largely preserved. He lost even all information related to his personal identity including his own and parents' names, birth date, and place of birth. Although the possibility that he suffered from head injury when the earthquake attacked him cannot be excluded, no obvious findings indicative of previous brain trauma were detected by brain MRI. Physical and neurological examinations excluded any other somatic problems associated with organic amnesia [2, 5]. He is fully alert, and no psychiatric disorders associated with amnesic symptoms [2] are indicated. Malingering is unlikely because of lack of intelligible reward he could obtain from simulation for such a long period. Instead he has been eager to know his personal identity and life history. These considerations let me diagnose the patient as functional or psychogenic amnesia [1, 2, 6–8].

The differentiation of psychogenic from organic amnesia is not always easy, as both may be combined [5]. Recently, this dichotomy has been criticized [9, 10], and many researchers refer to the term “functional” to indicate the block of a function, triggered by either physical or psychic trauma [11].

The retrograde amnesia of this patient was presumably triggered by a natural disaster, the Great East Japan Earthquake in 2011, from which more than 15,000 people died and more than 2,500 were missing. Posttraumatic stress disorder (PTSD) gains much attention as psychological reaction in the aftermath of such disaster [12]. The patient also suffers from emotional and/or behavioral symptoms characteristic of PTSD, such as intrusive thoughts, flashbacks, and nightmares. It has been reported that many veterans of the Iraq-Iran war suffering from PTSD symptoms demonstrate comorbid dissociative disorders including psychogenic amnesia [13].

The course of functional amnesia is varied, though most patients recover their missing memories sooner or later. However, some patients remain in the same condition as the time of onset of amnesia for more than several years [8, 14], as in the present case. At present, it is unpredictable whether this patient regains his personal identity and lost episodic-autobiographical memory when he faces his family members and objective facts associated with his life history after identification of his personal background.

## 4. Conclusion

Although it is uncertain what event(s), physically and/or psychologically, associated with the huge earthquake and subsequent tsunami, predisposed this patient to amnesia, he has been suffering from long-lasting pure retrograde episodic-autobiographical memory loss covering his whole lifetime, with impaired self-identification. I have not been aware of medical reports on other cases suffering from such profound and persistent amnesia associated with this natural disaster. Even if this patient is an exceptional case, there might be other patients suffering from dissociative symptoms such as fugue, depersonalization, and amnesia [15]. Most of such cases are probably ignored as subthreshold in the catastrophic situations in the aftermath. Physicians and other professionals who take care of victims of disaster should be aware of such dissociative spectrum disorders [16].
